# Some Things about Rain

**Published:** 1875-01

**Authors:** 


Some Things About Rain.—We have seen that the amount of watery vapor that the air can hold depends upon its temperature. When saturated with moisture, it must give up a portion of it, if the temperature falls. Whatever cools the air may, therefore, be considered as a cause of rain. It is chiefly due to the ascent of air into the higher parts of the atmosphere by colder, drier, and therefore heavier wind currents which get beneath them. Ranges of mountains also oppose their masses to the winds, so that the air forced up their slopes is cooled, and its vapor condensed into showers of rain or snow. The air as it rises is chilled by its expansion as well as by the lower temperature of the region into which it ascends. The deposition of vapor on the sides of a glass receiver which is being exhausted by an air-pump is a familiar illustration of this fact. The air is cooled as it becomes rarefied, and has to yield up a part of its vapor.

The temperature of the air is also lowered, and the rainfall increased, by winds that convey the air to higher and colder latitudes. This occurs in temperate regions, or in those traversed by the return trade-winds, which north of the equator blow from the southwest, and south of it from the northwest. The meeting and mixing of winds of different temperatures is also a cause of rain, since the several portions when combined cannot hold as much vapor as before. The rainfall is also increased if the prevailing winds are directly from the sea, and therefore moist; but it is diminished if they have passed over large tracts of land, particularly mountain-ranges, and are therefore dry. The quantity of rain is likewise influenced by sandy deserts, which allow radiation, by day or night, to take immediate effect in raising or depressing the temperature; and also by forests, which retard or counteract radiation.

Of the agencies that affect the distribution of the rainfall, the most important are mountains. These, as we have remarked, act as condensers of the atmospheric vapor, and air-currents that reach them heavy with moisture are plundered of their watery treasures as they climb the rocky slopes, and have little or nothing left for refreshing the thirsty plains on the farther side. In the article to which we have referred above, it was stated that there are some regions where rain rarely or never falls; as the coast of Peru, the Sahara in Africa, and the desert of Gobi in Asia. All these rainless districts are made so by the mountains on their borders.

The Sahara is bounded on the north and on the south by ranges of mountains. When the northeast trade-wind strikes the northern range, a part of its vapor is condensed. As it moves southward, it reaches warmer latitudes, where there is a greater capacity for moisture. Since there are no opposing winds to force it upwards, it sweeps on across the vast sandy plain until it arrives at the southern mountains, where its vapor is precipitated in abundant rains. In the few spots in the desest where hills or mountains occur, there are occasional rains. On the desert of Gobi, the prevailing winds are from the southeast, and are very dry, because they have precipitated all their moisture in passing over the Himalaya Mountains. The rainless district in Peru is caused by the Andes, which condense nearly all the vapor of the southeast trade-wind in copious rains on their eastern slopes.

The unequal distribution of rain in the British Isles is another illustration of the action of mountains as condensers of the atmospheric vapor. Tyndall, in his "Forms of Water," thus refers to the heavy rainfall in the southwestern part of Ireland:

"Imagine a southwest wind blowing across the Atlantic towards Ireland. In its passage it charges itself with aqueous vapor. In the south of Ireland it encounters the mountains of Kerry; the highest of these is Macgillicuddy's Reeks, near Killarney. Now, the lowest stratum of this Atlantic wind is that which is most fully charged with vapor. When it encounters the base of the Kerry Mountains, it is tilted up and flows bodily over them. Its load of vapor is therefore carried to a height, it expands on reaching the height, it is chilled in consequence of the expansion, and comes down in copious showers of rain. From this, in fact, arises the luxuriant vegetation of Killarney; to this, indeed, the lakes owe their supply. The cold crests of the mountains also aid in the work of condensation.

"Note the consequence. There is a town called Cahirciveen, to the southwest of Macgillicuddy's Reeks, at which observations of the rainfall have been made; and a good distance farther to the northeast, right in the course of the southwest wind, there is another town, called Portarlington, at which observations of rainfall have also been made. But before the wind reaches the latter station, it has passed over the Mountains of Kerry and left a great part of its moisture behind it. What is the result? At Cahirciveen the rainfall amounts to 59 inches in a year, while at Portarlington it is only 21 inches."

The irregularities in the rainfall of the English "Lake District" are to be similarly explained. The mountains of Cumberland, like those of Kerry, act as condensers of the moisture brought by the southwest winds, so that the average rainfall at different points in their vicinity is from 90 to 180 inches, while in the east of England it is not more than 20 to 28 inches.

The heaviest rainfall known, as we have before stated, is on the Khasia Hills in British India. It averages 600 inches annually, 500 inches of which fall in seven months of the year. This extraordinary opening of the windows of heaven is yet another example of mountain condensation. These hills face the Bay of Bengal, from which they are separated by only 200 miles of swamps and marshes. Hence, the southerly winds not only arrive heavily laden with vapor from the Indian Ocean, but they get more moisture in passing over the 200 miles of swamp. They are therefore ready to burst in torrents, even before they are suddenly raised, by the hills they encounter, into the cooler regions of the atmosphere.

At places within the tropics, where the trade-winds blow regularly and steadily, the rainfall is small. Since these winds come from higher latitudes, the temperature is increasing, and they are thus more likely to take up moisture than to part with it. Where, however, they are forced up the slopes of mountain ranges, they bring abundant rains.

The tropical belt known as "the region of calms" is the region of constant rains. Here the sun almost invariably rises in a clear sky, but about midday clouds rapidly gather over the whole face of the sky and pour down prodigious quantities of rain. Towards evening the clouds disappear, the sun sets in a clear sky, and the nights are serene and fine. The reason of this is that the air, being greatly heated by the vertical rays of the sun, ascends, drawing with it all the vapor which the trade-winds have brought with them, and which has been largely increased by the rapid evaporation from the belt of calms; and this vapor is condensed as it rises. The rain is sometimes so copious that fresh water has been collected from the surface of the sea. As evening sets in, the surface of the earth and the air near it being cooled, the ascending currents cease, and the cool air descends; the clouds are thus dissolved, and the sky continues clear till the returning heat of the following day.

Over a great part of the tropics, disturbing influences draw the trade-winds out of their course, and sometimes, as in the case of the monsoons, give rise to winds from the opposite point of the compass. But for these winds the eastern district of Hindostan would be constantly deluged with rain, and the western districts constantly dry and arid. As it is, each part of India has its dry and wet seasons, summer being the wet season of the west and interior as far as the Himalayas, and winter the wet season of the east, and especially the southeast.

We have lately met with a striking illustration of the effect that even a very slight elevation of land may have upon moist air-currents coming from seaward. At Brighton, England, there is a pier 1115 feet long, at the end of which the rainfall for the year 1872 was only 23 inches, while at a point about 1100 feet inland from the head of the pier it was 38.52 inches—a difference of 15.52 inches at stations less than half a mile apart. For the year 1873 the figures were 14.55 and 26.74, showing a difference of 12.19 inches. The observations were made by an experienced meteorologist, with the aid of the best rain-gauges, and their accuracy is unquestionable.—*Boston Journal of Chemistry.*

